# Individual size but not additional nitrogen regulates tree carbon sequestration in a subtropical forest

**DOI:** 10.1038/srep46293

**Published:** 2017-04-20

**Authors:** Jianping Wu, Honglang Duan, Wenfei Liu, Xiaohua Wei, Yingchun Liao, Houbao Fan

**Affiliations:** 1 Jiangxi Key Laboratory for Restoration of Degraded Ecosystems & Watershed Ecohydrology, Nanchang Institute of Technology, Nanchang, Jiangxi 330099, China; 2 Department of Earth, Environmental and Geographic Sciences, University of British Columbia, 3333 University Way, Kelowna, B.C., V1V 1V7, Canada

## Abstract

Recent studies have indicated that tree carbon accumulation in subtropical forests has been negatively affected by global change phenomena such as warming and drought. However, the long-term effect of nitrogen addition on plant carbon storage remains poorly understood in these regions. In this study, we conducted a 10-year field experiment examining the effect of experimental N addition on plant growth and carbon storage in a subtropical Chinese fir forest. The N levels were 0 (control), 60, 120, and 240 kg ha^−1^ yr^−1^, and the N effects on tree carbon were divided into stand and individual levels. The results indicated that tree carbon storage at the stand scale was not affected by long-term N addition in the subtropical forest. By contrast, significant impacts of different tree size classes on carbon sequestration were found under different N treatments, which indicated that the amount of plant carbon sequestration was significantly enhanced with tree size class. Our findings highlight the importance of community structure and growth characteristics in Chinese fir forests, in which individual size but not additional N regulates tree carbon sequestration in this subtropical forest.

Global atmospheric nitrogen (N) deposition has dramatically increased due to anthropogenic activities over the past century^1^. Along with continued fossil-fuel burning and artificial fertilizer application, N deposition has been predicted to increase further in many areas around the world^2^. In China, rapid economic development, such as industrialization and urbanization, has been accompanied by large amounts of N emission during recent decades^3^; annual reactive N inputs into the atmosphere have increased from 7.6 to 20 Tg from 1978 to 2010^4^, and annual bulk N deposition has increased by approximately 8 kg N per hectare between the 1980s and the 2000s^3^. Therefore, increasing N deposition in China has caused great concern because of its potential effects on different ecosystems^5^,^6^.

One of the most interesting topics is how increased N deposition influences global carbon (C) cycling or atmospheric CO_2_ concentrations, particularly the effects of N deposition on forest carbon dynamics. Forest areas occupy nearly 4.0 billion ha of the Earth’s terrestrial surface^7^ and are considered to be a large C sink that is estimated to sequester 2.4 Pg C per year^8^. Many studies using models^9^,^10^ and meta-analysis^11^,^12^ have indicated that N deposition promotes forest carbon storage and plays an important role in mitigating anthropogenic CO_2_ emissions. At a regional scale, there have been reports indicating that N deposition promotes ecosystem carbon sequestration in temperate forests^13^,^14^,^15^. Furthermore, a meta-analysis indicated that N deposition impedes the decomposition of soil organic matter and thus improves carbon storage in temperate forests^16^. In tropical or subtropical forests, however, the effects of N deposition on forest carbon storage depend on forest characteristics such as soil nutrients and latitude^6^,^17^.

Based on the stand level, long-term experiments have investigated the effects of N deposition on above- and belowground carbon dynamics in temperate or boreal regions. For example, a 30-year N loading experiment initially improved tree growth, and then the amount of N addition regulated the growth rate in a boreal forest^18^. In tropical or subtropical regions, however, few studies of N addition in forests have been longer than 10 years^19^,^20^, generating difficulty in assessing the effect of nitrogen deposition on forest carbon cycling^16^. In tropical and subtropical forests^17^,^21^, N addition usually promotes forest carbon sequestration, and the increased carbon sequestration has been mainly ascribed to soil carbon but not plant growth^22^,^23^. However, a minimal impact from N deposition on plant carbon has also been found in temperate and boreal forests^24^,^25^. For instance, N addition did not significantly affect woody biomass increments for five tree species in Northeastern America^26^.

The potential mechanism of plant non-response was attributed to the N saturation or N-induced soil acidification in these forest ecosystems^26^,^27^. Most of these arguments were based on the stand level, and the effects of individual trees were not considered. However, the size of individual trees would play different roles in a forest and result in different responses to environmental change^28^,^29^. For example, evergreen broad-leaved forests in China are undergoing a change from forests that are dominated by a cohort of fewer and larger individuals to forests dominated by a cohort of more and smaller individuals in response to global warming and drought stress^30^, and the biome-scale reconstitution of forests would strongly influence the regional carbon dynamics^31^. Although there were several N addition experiments concerning tree growth more than 10 years long^14^,^18^,^20^, as far as we know, no study has used long-term (>10 years) manipulation experiments to investigate the responses of individual trees to N addition in China. It is unclear whether unchanged plant growth after N deposition is affected by different growth dynamics among different tree sizes.

In this article, we used data from a10-year observation plot in a Chinese fir forest to examine whether N addition affects net primary production at the stand level and whether the responses of plant carbon sequestration are affected by tree size. The Chinese fir (*Cunninghamia Ianceolata*(Lamb.)Hook.) has been widely planted in 14 provinces and is one of the most important commercial forest species in subtropical China^32^,^33^. It has been reported that the total area of Chinese fir plantations was approximately 1.12 × 10^7^ ha in 2009, which accounted for 18.17% of all reforested plantations in China^34^. We hypothesized that N treatments have minimal impacts on tree carbon sequestration but that carbon sequestration in trees of different sizes would vary in their responses to N addition. Furthermore, we also predicted that tree size would play a greater role in carbon storage overtime due to its growth characteristics.

## Results

### Plant response at the stand level

**Figure 1:**
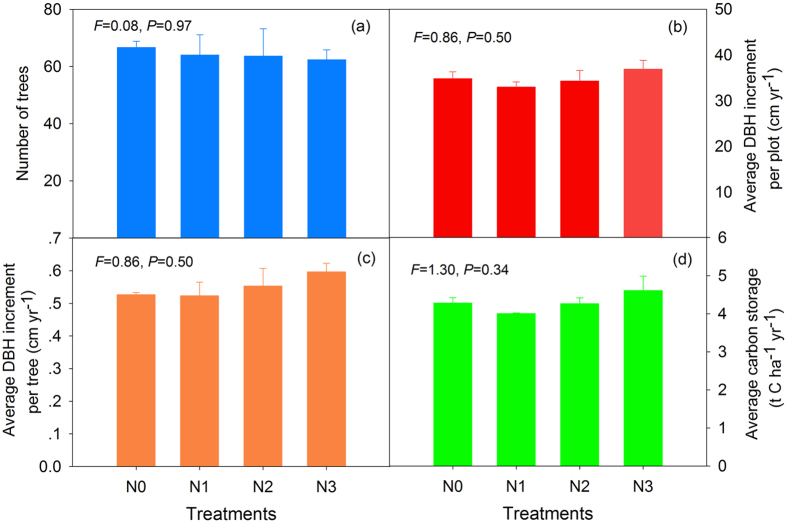
The number of trees (**a**), average DBH increment per plot (**b**), and average diameter at breast height (DBH) increment per tree (**c**) and average carbon sequestration (**d**) after 10 years of N addition. Values are the means ± SEs of the three plots. Within each panel, the *F*-value and *P*-value are shown based on a one-way ANOVA.

The tree diameters were normally distributed prior to and after N addition. The diameter of most individual trees was from 10 cm to 25 cm before N addition (Supplementary Figure 1), whereas the diameter increased from 15 cm to 30 cm after a decade of N treatment (Supplementary Figure 2). The tree numbers in the four treatments were similar, averaging 66 per plot (Fig. 1a).The average DBH increments ranged from 32.96 cm to 36.88 cm, but there was no significant difference among treatments (Fig. 1b). When we divided by tree number in each plot, the average DBH increments were 0.55 cm per tree per year during the 10-year treatment, and they were also not statistically significant (Fig. 1c). The average carbon storage per year was similar to the DBH increment (Fig. 1d).

### Individual plant response

**Figure 2:**
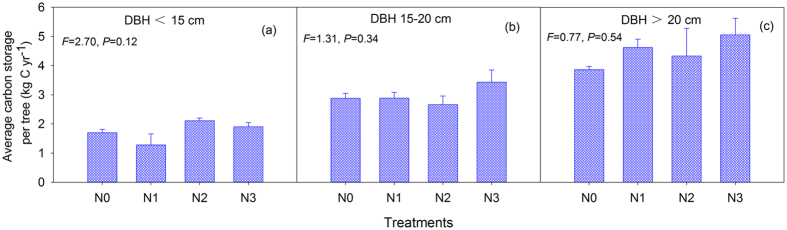
The average carbon storage per tree per year among N treatments for the three DBH classes (DBH < 15 cm, 15 cm ≤ DBH ≤ 20 cm and DBH > 20 cm). The values are the means ± SEs of the three plots. Within each panel, the *F*-value and *P*-value are shown based on one-way ANOVA.

**Figure 3:**
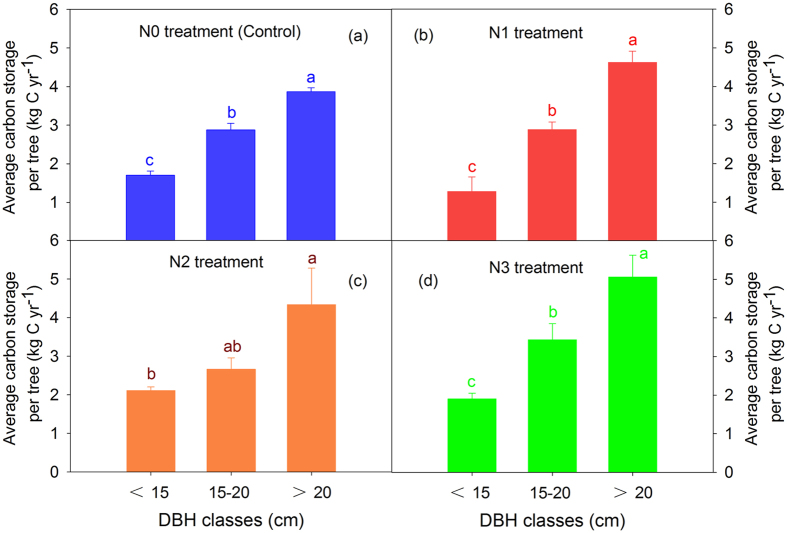
The average carbon storage per tree per year among the tree sizes for the four N addition treatments. The values are the means ± SEs of the three plots. Within each panel, the means with different letters are significantly different based on ANOVA and LSD (*P* < 0.05).

**Figure 4:**
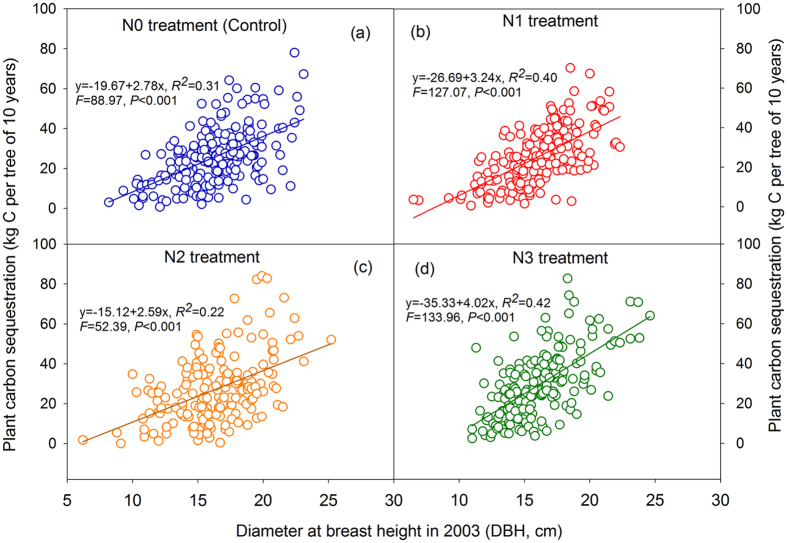
A regression between plant carbon sequestration and the diameter at breast (DBH) in the four N treatments. The plant carbon sequestration represents the difference before and after N treatment after a decade. The DBH represents the value prior to N treatment.

The individual DBH increments in the three classes from 2003 to 2013 did not exhibit an obvious increasing trend among the N treatments, although significant differences were detected at 15 cm ≤ DBH ≤ 20 cm (Supplementary Figure 3). The similar trends of decreased tree numbers at DBH < 15 cm and 15 cm ≤ DBH ≤ 20 cm, and the increased tree numbers at DBH > 20 cm, were found after 10 years of N addition (Table 1). The average carbon storage per tree per year was not significantly different among N treatments for the three DBH classes (DBH < 15 cm, 15 cm ≤ DBH ≤ 20 cm and DBH > 20 cm) (Fig. 2). By contrast, if we calculated the average carbon storage per tree per year among tree sizes, the results exhibited an obvious increasing trend of carbon storage with tree size (Fig. 3). A regression analysis of the plant carbon sequestration with the DBH showed a strong positive relationship in the four N treatments (Fig. 4). The two-way ANOVA also indicated that the DBH class but not N addition significantly affected the carbon storage (Table 2).

## Discussion

Our data indicated that N addition did not affect plant carbon storage after 10 years of treatment at the stand level. Similar results were reported in our previous study, which showed that plant carbon sequestration did not respond to N addition or to the interaction between sampling year and N addition over a shorter time scale^23^. The secondary forests or early successional forests were usually grouped into N-limited ecosystems^6^,^35^, where experimental N fertilization would promote plant carbon accumulation^17^. For example, Chen *et al*.^6^ reported that N addition increased the aboveground plant carbon pools, especially in N-limited subtropical forests. The lack of positive responses in plant carbon sequestration after N addition would be attributable to N saturation at our study site. A high background of N deposition in South China has been reported^3^,^4^. In fact, many studies of temperate or tropical forests have reported that when an ecosystem reaches N saturation, plant growth or aboveground net primary production would not be improved by N addition^22^,^26^,^36^. In addition, the soil available N significantly enhanced along with N addition^37^, which was also consistent with Chen *et al*.’s report that N addition caused further N saturation in humid tropical forests^38^. The second factor was co-limitation by other nutrients. For example, phosphorus is considered to be a limiting factor for plant growth in tropical regions^39^. Although N addition alone did not increase plant carbon accumulation, the combined addition of N and other nutrients significantly facilitate tree growth in tropical forests^19^,^20^. The contents of available phosphorus had not the same trend with soil available N after N addition^37^, which partially supported the assertion. Third, the three DBH classes had no responses to the four levels of N deposition, which consequently might have limited variations in carbon sequestration among them. Our results agree with a previous study conducted in tropical forests that found no effect of N addition on plant diameter increments for different tree size classes^22^.

Furthermore, it is worth noting that the marginal effect of N deposition on plant growth in our study was supported by an assessment based on the FORECAST model^33^. When N deposition levels exceeded 20–30 kg ha^−1^ yr^−1^, N saturation in Chinese fir forests would occur and the incremental impacts of N deposition on forest carbon storage would not be obvious^33^, which is consistent with our results. The marginal response of tree growth to long-term N addition would suggest that N deposition has a small negative effect on subtropical forests. It is well known that ecological functions and services are important in forests in the context of climate change^8^. In Chinese monsoon evergreen broad-leaved forests, global warming and drought stress resulted in a decline of mean DBH and in larger individual trees accompanied by more and smaller individuals, which suggests that ecosystem resilience is threatened by long-term climate change in subtropical forests^30^. However, at least from our study, the subtropical Chinese fir plantations would not be strongly affected by long-term N addition.

Interestingly, when comparing plant carbon storage in different tree size classes, we found that plant carbon sequestration was significantly different. The bigger tree size classes sequestered larger amounts of carbon in our study. Recent research has indicated that individual tree size plays an important role in ecosystem carbon storage^28^, which is consistent with our results. For instance, large trees do not only act as senescent carbon reservoirs but also assimilate larger amounts of carbon compared with smaller individuals^28^. In a previous study from tropical forests, it was also reported that the incremental rate of tree diameter growth was significantly higher for larger size classes than smaller size classes, and no effect of fertilization for any tree size class^22^. We realized that these arguments were mainly based on the studies from broad-leaved forests, which may be different from coniferous forests. They further highlighted a critical need in future studies to investigate the responses of different tree sizes to N addition and determine the mechanisms involved in coniferous forests.

In conclusion, our long-term investigation suggests that tree carbon storage would not be affected by N deposition in Chinese fir forests. Further, N addition had no effect on carbon sequestration in different tree size classes. Plant carbon sequestration was significantly enhanced with tree size class, which indicated the importance of community structure and growth characteristics in the subtropical forest. We also suggest that more attention should be paid to investigating belowground processes, such as the soil biota community and soil carbon dynamics in response to N deposition, due to their large potential carbon pool based on our current and previous studies^23^,^40^.

## Materials and methods

### Site description

The study area was located at the Guanzhuang National Forestry Farm (117°43′E, 26°30′N), in Sanming City, Fujian Province, South China. The climate of this region is a typical subtropical monsoon climate with mean annual precipitation of 1606–1650 mm and a mean annual temperature of 18.8–19.6 °C. The soil is classified as an acrisol. The selected Chinese firs in this study site were planted in 1992 at a density of 1660 trees per ha over a total of 5173 ha. This long-term experiment was initiated in December 2003 when the plantations were 12 years old. The plantations were established on hilly land with uniform site characteristics. The initial characteristics of the plantations and soil in the N addition plots in December 2003 were reported in our previous papers^23^,^41^.

### Experimental design

We randomly established 12 experimental plots over a 6 ha section of the plantation. Each plot was 20 m × 20 m and was treated with one of four levels of N. The treatment codes and levels (kg N ha^−1^ yr^−1^) were N0 (0), N1 (60), N2 (120), and N3 (240). The plots were randomly arranged and each treatment had three replicate plots. For each treatment and each plot, the required amount of urea [CO(NH_2_)_2_] was dissolved in 20 L of tap water, and the solution was sprayed onto the soil surface every month beginning in January 2004. The control plots received an equivalent volume of water without CO(NH_2_)_2._

### Investigation of plant growth, carbon storage, and litter input

In total, 796 trees were investigated from all of the plots. The tree diameters at breast height (DBH at 1.3 m point) were measured at the start of the experiment in 2003 and at the end of 2013. For the carbon storage of each tree, the allometric relationship between DBH and tree biomass was fitted with a power function by our research group^23^, which was Biomass = 0.48 × DBH^1.84^ (r^2^ = 0.91, *P* < 0.001, n = 12). The proportion of carbon in the dried plant biomass was assumed to be 0.45. We first calculated tree growth and carbon sequestration at the stand level. To identify the response of tree size to N deposition, we divided all individuals into three DBH classes (DBH < 15 cm, 15 cm ≤ DBH ≤ 20 cm and DBH > 20 cm). We classified each tree into one of those three classes based on the DBH data of the first survey in December 2003. The last surveys were conducted in December 2013. The annual average growth or carbon storage = (the values from 2013 - the values from 2003)/10. Only 1% of trees died in our study site during the studied period and they were considered as no growth when analysis was conducted.

### Statistical analyses

One-way analyses of variances (ANOVAs) were used to analyse the effect of treatment (levels of N addition) or tree size (DBH classes) on tree growth and carbon storage. Two-way ANOVAs were used to determine the effects of N deposition and tree size on individual plant carbon storage. SPSS 15 (SPSS Inc., Chicago, IL, USA) was used for the statistical analyses. Differences were considered significant at the 0.05 level. The data for tree DBH and plant carbon sequestration was fitted with linear regression: y = ax + b, where a and b were two parameters. All fittings were conducted using regression function in Sigmaplot 12.0 (Systat Software Inc., San Jose, CA, USA).

## Additional Information

**How to cite this article:** Wu, J. *et al*. Individual size but not additional nitrogen regulates tree carbon sequestration in a subtropical forest. *Sci. Rep.* **7**, 46293; doi: 10.1038/srep46293 (2017).

**Publisher's note:** Springer Nature remains neutral with regard to jurisdictional claims in published maps and institutional affiliations.

## Supplementary Material

Supplementary Figures

## Figures and Tables

**Table 1:** Changes in actual tree numbers in three classes from 2003 to 2013.

	N0		N1		N2		N3	
	2003	2013	2003	2013	2003	2013	2003	2013
<15 cm	64	21	69	17	56	13	69	9
15–20 cm	120	51	108	61	113	58	101	59
>20 cm	18	130	16	115	24	122	18	120

The DBH classes were defined at the beginning of the experiment, which remained in that class even if it grew large enough to enter a larger DBH class.

**Table 2:** *F* and *P* values of the effects of diameter at breast height (DBH), N treatment, and their interaction on the average carbon sequestration per tree per year after 10 years of N treatments.

Source	*df*	Sum of Square	Mean Square	*F*	*P*
DBH class	2	4464.63	2232.32	48.93	** < 0.001**
Nitrogen	3	216.822	72.27	1.58	0.22
DBH × Nitrogen	6	218.823	36.47	0.80	0.58
Error	24	1094.93	45.62		
