# Supplementary material for: Individual size but not additional nitrogen regulates tree carbon sequestration in a subtropical forest
Source: Sci Rep. 2017 Apr 20;7:46293. doi: 10.1038/srep46293 (PMC5397863; doi:10.1038/srep46293)
Supplement: Supplementary Figures [file srep46293-s1.pdf]

Title: Individual size but not additional nitrogen regulates tree carbon sequestration in a subtropical forest

Authors: Jianping Wu[1], Honglang Duan[1], Wenfei Liu[1], Xiaohua Wei[2], Yingchun Liao[1], Houbao Fan[1,*]

Affiliations: [1]Jiangxi Key Laboratory for Restoration of Degraded Ecosystems & Watershed Ecohydrology, Nanchang Institute of Technology, Nanchang, Jiangxi 330099, China

[2]Department of Earth and Environmental Sciences, University of British Columbia, 3333 University Way, Kelowna, B.C., Canada V1V 1V7

*** Corresponding author and reprint requests:**

Dr. Prof. Houbao Fan,

Jiangxi Key Laboratory for Restoration of Degraded Ecosystems & Watershed Ecohydrology, Nanchang Institute of Technology, Nanchang, Jiangxi 330099, China

E-mail address: hbfan@nit.edu.cn, Tel.: +86 79182085311, Fax: +86 791 88126632

Type of manuscript: Regular article **Date of resubmission:** Dec 28, 2016

Number of text pages: 23 **Number of words:** 4176

Number of tables: 2 **Number of figures:** 5

Running Title: Individual size affects tree carbon storage

S-Figure 1. Histograms of frequency distribution of individual size in the four treatments in 2003.

S-Figure 2. Histograms of frequency distribution of individual size in the four treatments in 2013.

S-Figure 3. Boxplots of DBH increment with three classes from 2003 to 2013. Within each panel, *F*-value and *p*-value are shown based on one-way ANOVA.#

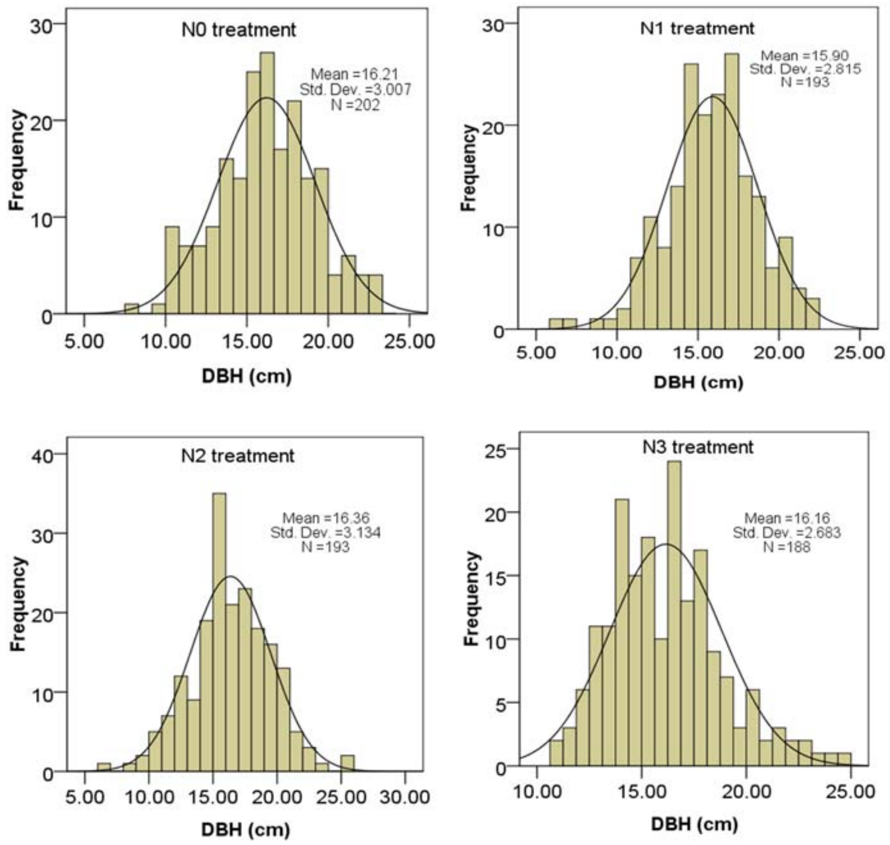

S-Figure 1

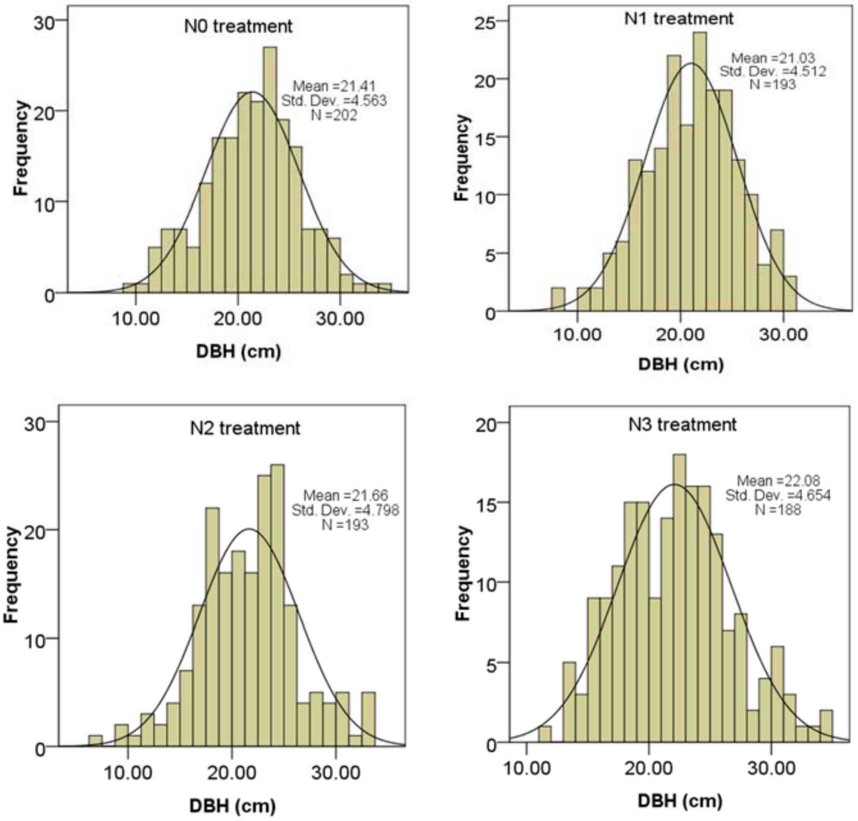
N0 treatment
Mean =21.41
Std. Dev. =4.563
N =202
Frequency
DBH (cm)
N1 treatment
Mean =21.03
Std. Dev. =4.512
N =193
N2 treatment
Mean =21.66
Std. Dev. =4.798
N =193
N3 treatment
Mean =22.08
Std. Dev. =4.654
N =188

S-Figure 2

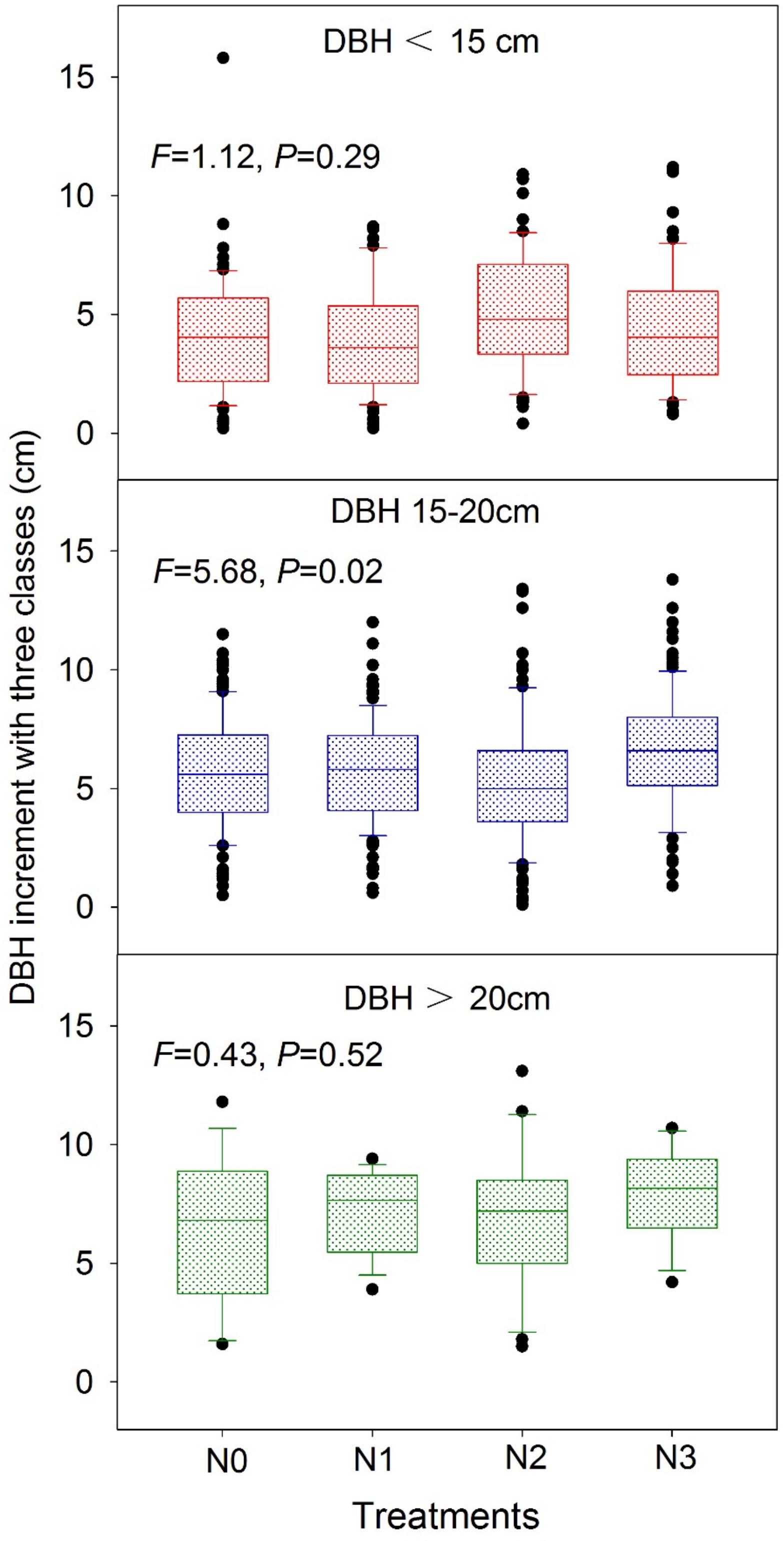
DBH ＜ 15 cm
F=1.12, P=0.29
DBH 15-20cm
F=5.68, P=0.02
DBH ＞ 20cm
F=0.43, P=0.52
DBH increment with three classes (cm)
0
5
10
15
N0
N1
N2
N3
Treatments

S-Figure 3
